# Endoscopic Ear Surgery, from the Last Ten to the Next Ten Years: A Critical Perspective

**DOI:** 10.3390/jcm13216300

**Published:** 2024-10-22

**Authors:** Matteo Alicandri-Ciufelli, Giulia Molinari, Edoardo D’Alessandro, Riccardo Nocini, Livio Presutti, Daniele Marchioni

**Affiliations:** 1 Department of Otolaryngology and Head and Neck Surgery, University Hospital of Modena, 41121 Modena, Italy; 2 Department of Otolaryngology-Head and Neck Surgery, IRCCS Azienda Ospedaliero-Universitaria di Bologna, 40138 Bologna, Italy; 3 Alma Mater Studiorum-Università di Bologna, 40138 Bologna, Italy; 4 Department of Otolaryngology and Head and Neck Surgery, University Hospital of Verona, 37134 Verona, Italy

**Keywords:** ear surgery, endoscope, endoscopic ear surgery, endoscopy, lateral skull base, middle ear, transcanal

## Abstract

Endoscopic ear surgery has gained increasing popularity starting from the early 2000s, introducing new anatomical, physiological and surgical concepts in ear and lateral skull base surgery. Its development has brought new scientific knowledge, thus improving surgical technique with a minimally invasive attitude. The aim of this perspective is to review and summarize all the steps that brought endoscopic ear surgery from a surgery practiced by a few teams to a worldwide recognized advancement in otology.

## 1. Introduction

Endoscopic ear surgery has gained increasing popularity starting from the early 2000s, introducing new anatomical, physiological and surgical concepts in ear and lateral skull base surgery. From being practiced and sponsored by a few teams, it is nowadays a recognized reality all over the world. In particular, during the last ten years, scientific validation and long-term results have been published, contributing to its success. New indications and technical refinements have been introduced, although the continuous technological advancements make it a fertile ground for further potential improvements in the coming years. The aim of the present perspective is to illustrate the main goals that have been reached and potential future advancement based on the most up-to-date literature.

## 2. Material and Methods

A narrative literature review was performed. The following search strings were used on PubMed: “endoscopic ear surgery” OR “endoscopic lateral skull base surgery”. The results were implemented by a manual search from references. The most relevant articles were included, analysed and discussed.

## 3. Results

The search strings documented an increasing interest in the international literature, testified to by the number of publications on the topic of endoscopic ear surgery, which went from fewer than 10 in the decade 1990–2000 to more than 100 in the decade 2000–2010, and to more than 400 between 2010 and 2020. The same number of articles (~400) were published between 2020 and 2024. The publications covered an increasing range of topics, from the pure anatomical recognition of the first experiences, to the most advanced procedures on the endoscopic lateral skull base and intradural neurosurgery.

## 4. Discussion

### 4.1. The Beginnings of Endoscopic Ear Surgery (EES)

The first authors to document endoscope application to the middle ear described cholesteatoma surgery in 72 patients [1,2], concluding that endoscopy is an essential complement to the surgical microscope to control some regions of the middle ear such as the sinus tympani. Other authors suggested the use of the endoscope through the mastoidectomy cavity in second-look procedures [3]. The instrumentation was derived from nasal surgery and was based on rigid endoscopes (4mm and 2.7 mm) with different lengths and different angles of view (0, 30, 45, 70°), as well as a camera and monitor system. At that time, endoscopes were used as an adjunct to microscopes to check possible residual pathologies in hidden areas, but not as an exclusive technique. In 1992, the first case series of patients treated by endoscopic myringoplasty was published [4], reporting a success rate higher than 90%. In 1997, the first case series of 36 patients operated on by a totally endoscopic technique for attic cholesteatoma, without the aid of an operating microscope, appeared in the literature [5], concluding that endoscopic ear surgery (EES) was a safe and effective transcanal alternative to postauricular procedures. During the following years and the early 2000s, a few other case series were published, mainly describing combined (microscopic–endoscopic) techniques [6,7,8,9]. In the meantime, the use of exclusively EES for cholesteatoma treatment became more validated, with more case series and longer-term follow-ups [10]. Endoscopic approaches started to also raise interest for more specific applications, like explorative tympanotomies for conductive hearing loss [11].

### 4.2. Revisiting Middle Ear Anatomy

Mainly after 2010, the ability to look around the corner, the improvement in optics with the introduction of 3 mm 15 cm rigid endoscopes and the introduction of HD cameras allowed an increased interest in revisiting middle ear anatomy from an endoscopic perspective. These anatomic studies were carried out both on living patients and on cadavers.

First of all, endoscopy allowed a better understanding of the anatomy of the tensor fold area. Traditionally, microscopic approaches showed several limitations in reaching this structure, since, for example, in the canal wall up technique it is hidden mainly by the malleus. The use of an endoscope was demonstrated to be an effective way to explore this area and to point out the angulation and the complete/incomplete structure of this membranous fold. Using a 45° endoscope in the protympanum, it is possible to observe the inferior surface of the tensor fold, which separates the anterior epitympanum from the supratubal recess [12].

**Figure 1:**
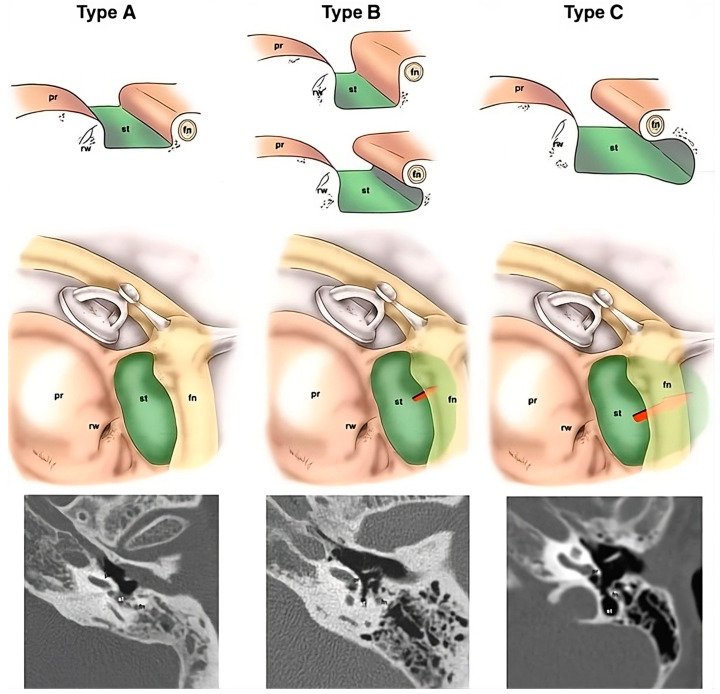
Classification of depth of the sinus tympani, based on an axial CT scan. Type A: small sinus tympani. Type B: deep sinus tympani. Type C: deep sinus tympani with posterior extension. Fn, facial nerve; pr, promontory; rw, round window; st, sinus tympani.

The use of an endoscope to explore the recesses of the retrotympanum is an anecdotal example of how this instrument allows for the evaluation of blind spots. Although Proctor in 1969 described the anatomy of the posterior tympanic wall and identified the sinuses of the retrotympanum [13], they were poorly described later in the literature because they were barely visible enough by microscope to properly assess their anatomic features. For example, even traditional microscopic approaches such as the canal wall down technique prevented the direct visualization of the sinus tympani, and the only way to indirectly explore this cavity was using small mirrors. Since the introduction of middle ear endoscopies, it has been possible to better describe the shape of this recess (classic/confluent/partitioned/restricted) and of other bony crests, such as the ponticulus and the subiculum and their anatomical variants. The depth of the sinus tympani has been classified into three types (A/B/C) according to its medial and posterior extension with respect to the facial nerve (Figure 1). Only sinus tympani types A and B can be completely explored using an endoscope [14,15].

**Figure 2:**
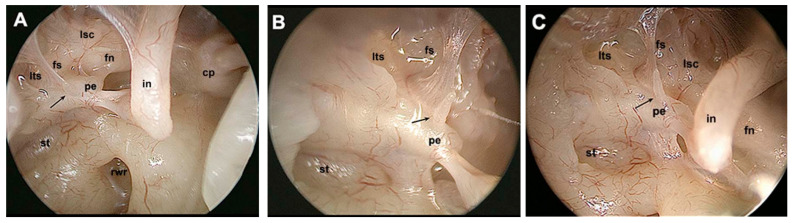
Endoscopic assessment of the facial sinus with differently angled endoscopes. (**A**): 0° endoscope. (**B**): 45° endoscope. (**C**): 70° endoscope. Black arrow indicates an incomplete chordiculus. Cp, cochleariform process; fn, facial nerve; fs, facial sinus; in, incus; lsc, lateral semicircular canal; lts, lateral tympanic sinus; pe, pyramidal eminence; rwr, round window region; st, sinus tympani.

In the same fashion, other retrotympanic structures were better described, such as the pyramidal eminence and the subpyramidal space. The latter has been described as an inconstant space lying medially to the medial aspect of the pyramidal eminence, more or less deep, directly communicating with the sinus tympani or with the posterior tympanic sinus or both, depending on the morphology of the ponticulus [16]. Endoscopy allowed for a better definition of other retrotympanic structures, such as the facial sinus (Figure 2). Similarly to the sinus tympani, a radiologic classification of the facial sinus was proposed, identifying types A/B/C, according to its medial and posterior extension with respect to the mastoid portion of the facial nerve. A radiologic–endoscopic correlation was proposed and angled endoscopes were needed to explore the facial sinus, particularly for the deeper ones. The chordal ridge that marks the inferior border of this sinus was described in its morphological variants and was renamed as the chordiculus, since it had a ridged shape in fewer than half of the temporal bones analysed [17].

Moving towards the inferior retrotympanum, endoscopes offered a better description of the so-called sustentaculum promontorii—renamed as the finiculus—whose shape can be bridge-like or ridge-like, or absent sometimes. The finiculus marks the border between the retrotympanum and hypotympanum. A sinus inferior to the sinus tympani had been identified in most patients and it was called the sinus subtympanicus [18].

The anatomy of the round window region, too, was better understood by the use of optics. The fustis, a smooth bone which forms the floor of the round window chamber, and the subcochlear canaliculus, a channel connecting the petrous apex to the middle ear cleft through a series of pneumatized cells, were described in detail, and their usefulness as landmarks during cochlear implant procedures was underlined [19].

Compared to a microscopic approach, endoscopes allowed for a better visualization of the protympanum, too, and this led to a better understanding of the anatomy of this area. The protympanic shape (triangular/quadrangular) can be properly assessed using angled optics. New anatomic structures such as the subtensor recess, the caroticocochlear recess, the protympanic spine and the protiniculum were defined. The subtensor recess was described and classified similarly to retrotympanic recesses in types A/B/C according to its depth. The protiniculum was described as a structure arising from the promontory and separating the hypotympanum from the protympanum. Different shapes were identified and described [20].

### 4.3. New Physiological Concepts Introduced

**Figure 3:**
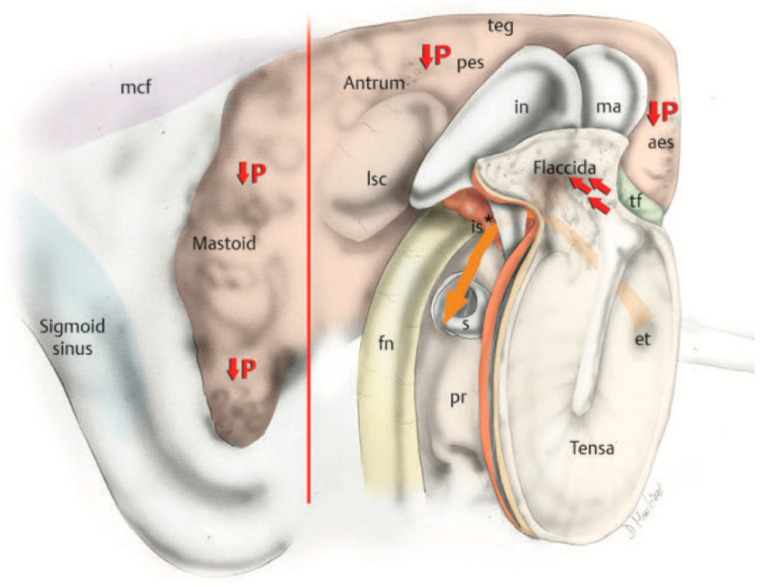
Posterior to anterior and lateral view of a pathological right ear after lateral epitympanic bone removal. The orange arrow indicates the direction of the air flow. An isthmus blockage (*) prevents air flow to the epitympanum, leading to pressure reduction inside the mastoid cavity and the epitympanum itself and consequently the creation of a retraction pocket of the tympanic membrane. Aes, anterior epitympanic space; et, Eustachian tube; fn, facial nerve; in, incus; is, isthmus; lsc, lateral semicircular canal; ma, malleus; pes, posterior epitympanic space; pr, promontory; teg, tegmen tympani; tf, tensor fold.

Better understanding of middle ear anatomy led to better comprehension of middle ear physiology. First, endoscopy played a key role in proving the “Selective Epitympanic Dysventilation Syndrome” [21], since it allows the surgeon to look around the corner without radically modifying the middle ear cleft anatomy. Previous studies from some of the present authors showed that besides normal Eustachian tube function, which has long been known as the main component of middle ear ventilation, a patent tympanic isthmus or an incomplete tensor fold are mandatory to ensure adequate epitympanic ventilation. Epitympanic retraction pockets and cholesteatomas have been associated with this pathogenetic mechanism: inadequate ventilation of the epitympanum determines pressure reduction in the epitympanic cavity and consequently retraction of the pars flaccida, with or without cholesteatoma development. Middle ear endoscopy allowed, moreover, a better comprehension of the anatomical–radiological correlations, since it was noted that the blockage of the epitympanic ventilation is associated with smaller volumes of epitympanic and mastoid cavities, compared to controls. Three patterns of ventilation route blockage were described: type A, described as a blockage of the isthmus associated with a complete tensor fold; type B, described as a blockage of the isthmus associated with a vertical blockage in the epitympanum, with or without a complete tensor fold; type C, described as a blockage of the isthmus associated with a complete tensor fold and a blockage of the antrum [22]. All this evidence allowed for a deeper comprehension of the physiology of middle ear ventilation, since chronic dysventilation syndrome causes the contraction of mastoid volumes, thus reducing its buffer effect (i.e., the capacity of “dilute” pressure changes inside the middle ear) and the mucosa surface available to transmucosal gas exchange [23] (Figure 3). These findings had a great impact on our everyday clinical practice, since restoring adequate ventilation of those cavities plays a central role during cholesteatoma surgery.

Middle ear endoscopic surgery allowed for a better understanding of middle ear vascularization, too.

During endoscopic procedures, in appropriate magnification and focus conditions, it was noted that movements of small red particles inside the vessels could be observed; this observation made it possible to determine the vessels’ blood flow direction. This fact, along with the possibility of almost completely checking the middle ear cavity without remodelling its anatomy during dissection, led to a better understanding of the anatomy and physiology of middle ear vascularization. Endoscopy allowed for the first in vivo study of the vascularization of this anatomic district. First, the vascularization of the promontory region and the incudostapedial region were studied. Despite identification of two almost constant vessels for each of these region (the inferior tympanic artery and the incudostapedial artery), a great variability in terms of morphology and blood flow direction was identified and consequently described. Even in the case of similar morphologies, it was possible to identify different directions of blood flow, varying from patient to patient. Apart from a better anatomical understanding of the middle ear cleft, these observations proved that the blood flow in the incudostapedial artery is directed from the incus towards the pyramidal eminence in most cases, in contrast to what was already described in the literature. This fact seemed to refute the usefulness of stapes tendon preservation in order to reduce the rate of lenticular process necrosis during stapedoplasty [24].

### 4.4. The Expansion of the Indications in Middle Ear Surgery

With the improvement of knowledge and technology, indications for endoscopic ear surgery expanded, starting after the 2010s [25]. In addition to 2.7 mm, 3 mm or 4 mm in diameter, several lengths were introduced, like 15 cm, 11 cm and 6 cm, allowing for better ergonomics and manoeuvrability. Three-chip and high-definition digital cameras attached to the endoscope, as well as high-definition digital monitors, further improved visualization, producing excellent quality images featuring automatic controls for white balance, colour and digital contrast enhancement. Also, bone drilling based on the piezoelectric ceramic handle contributed to the minimal invasiveness of the procedures by cutting bone while preserving soft tissues. Also, dedicated instruments sets, with suction integrated into the elevators and dissectors (e.g., the Panetti set), reduced the impact of bleeding during operations [26].

In 2013, for the first time some publications presented the results of endoscopic ear surgery in paediatric patients, also confirming the results in that population [26], and the benefits of avoiding postauricular incisions in younger populations [27].

The first experiences in stapes surgery were published in 2011 [28], demonstrating that endoscopic surgery is particularly suitable for stapedial disease, and can be performed even in patients with small and curved ear canals [29]. The anatomy of crucial regions during the procedure (such as the crus anterior, footplate in deep oval windows, chorda tympani insertion and pyramidal eminence) was visualized better than by microscope, improving confidence and results.

EES showed interesting applications in cochlear implants, too. The first case series of six patients was published in 2014. The endoscopic cochlear implant procedure allows for the positioning of the array, avoiding a mastoidectomy and posterior tympanotomy, thus potentially reducing complications for the facial nerve [30]. The visualization of the round window niche anatomy was deemed particularly useful in malformed or complex cases due to the anomalous course of the facial nerve, the anomalous conformation of the mastoid or the variations of the round window anatomy, in particular as an adjunct to the classic microscopic technique, or in difficult anatomical conditions and malformations [31].

Some technical refinements were also described for cholesteatoma surgery, introducing the endoscopic “open” tympanoplasty [32]. The endoscopic open techniques were indicated for sclerotic mastoid and attic cholesteatomas with antral involvement and provided a wide atticotomy to expose and completely exteriorize the antrum, which could be left open towards the external ear canal just as in classic microscopic canal wall down open techniques.

### 4.5. Endoscopic Lateral Skull Base Surgery

Lateral skull base (LSB) surgery represents the most recent field of surgery where the endoscopic technique found its application. The gradual integration of the endoscope into middle ear surgery led to expansion of the use of this technique to the inner ear, and then to the LSB.

The endoscope first entered the world of the LSB as an adjunct to the microscope during traditional approaches [33], thanks to the wide field of view and the availability of angled lenses. At the very beginning of the twenty-first century, for the first time in LSB surgery, the use of 30° and 45° lenses allowed surgeons to look around the corner of straight surgical fields dissected through the microscope and visualize most of the spaces considered to be difficult to access until that moment. This not only improved the understanding of the anatomy, but also enhanced the radicality of disease removal, reducing bone drilling in favour of a less invasive approach. The endoscope was previously used during retrosigmoid approaches for vestibular schwannomas, meningiomas and neurovascular conflicts, thanks to the angled view towards the IAC, which correlated with less bone drilling, enhanced dissection of the facial nerve and increased radicality of surgery [34,35,36]. Later on, reports on other endoscopic-assisted approaches were published, such as the infralabyrinthine, suprameatal translabyrinthine, transotic and infracochlear ones [37,38].

Parallel to the use of the endoscope as an additional tool during traditional microscopic approaches, exclusively endoscopic transcanal approaches were developed, based on the idea that the CPA is located at the end of an ideal line that runs from the external auditory canal to the internal auditory canal. Similarly, the suprageniculate area, the middle cranial fossa and the petrous apex could be reached through transcanal corridors running superiorly and inferiorly to the IAC.

Extensive research on human cadavers was performed to study the interface between the middle and inner ear from a transcanal perspective, in particular to identify the anatomical landmarks on the lateral and medial surfaces of the middle ear and the inner ear, and safely dissect the temporal bone, respecting the facial nerve, the internal carotid artery, the jugular vein and the dural sheets. The challenge was to understand the relationships between the anatomical structures seen from a different point of view, as compared to the traditional microscopic approaches.

Three main corridors to the lateral skull base were identified and proved to be successful for pathology removal involving the fundus, IAC, cochlea, petrous apex and geniculate ganglion region: the transcanal suprageniculate, the transcanal transpromontorial and the transcanal infracochlear corridor. Landmarks, tips and pitfalls of these approaches were refined over the years as clinical experience on patients was collected.

**Figure 4:**
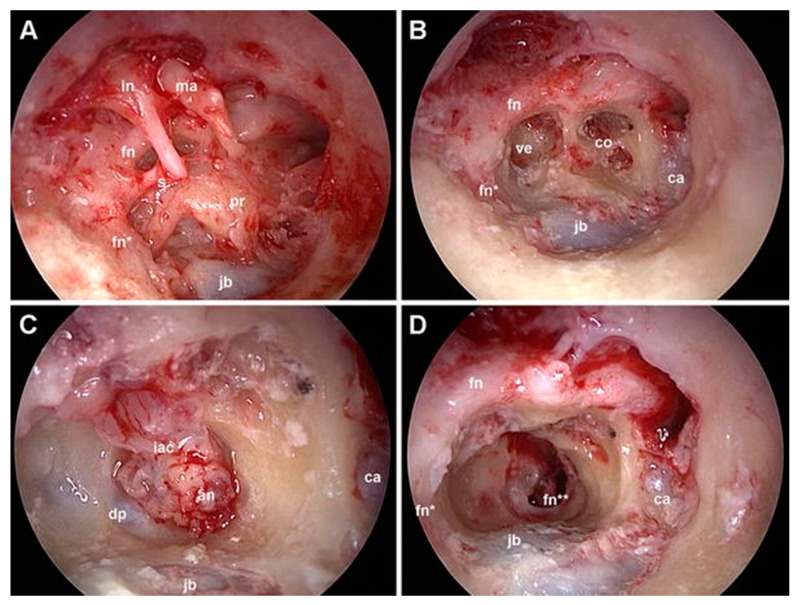
Exclusive endoscopic transcanal transpromontorial approach, right ear. (**A**): wide drilling of the external auditory canal is performed, thus exposing the tympanic cavity. (**B**): after ossicular chain removal and promontory drilling, the turns of the cochlea and vestibule are exposed. (**C**): the dissection proceeds, reaching the IAC; the dura is opened, showing in this case a vestibular schwannoma. (**D**): final tympanic cavity after acoustic neuroma removal with preserved facial nerve. In, incus; ma, malleus; s, stapes; ve, vestibule; ca, carotid artery; jb, jugular bulb; co, cochlea; fn, facial nerve (tympanic tract); fn*, facial nerve (mastoid tract); fn**, facial nerve (IAC segment); iac, internal auditory canal; an, acoustic neuroma; dp, reflection of the dura from the porus.

The very first clinical application occurred in 2013 with the treatment of a cochlear schwannoma via a transpromontorial approach. By 2017, surgeons began utilizing an exclusive transcanal transpromontorial approach for treating small vestibular schwannomas in the IAC. This technique involves accessing the IAC fundus via the promontory, allowing for the removal of disease within the vestibule, cochlea or IAC [39,40] (Figure 4).

After preliminary clinical reports, an expanded endoscopic–microscopic version of the approach was developed, namely the expanded transcanal transpromontorial approach (ExpTTA), to overcome the dimensional limits and the slow one-hand dissection of the endoscopic approach [41].

ExpTTA has been shown to be a safer alternative to the exclusive endoscopic technique, as it allows for a wider surgical field and better management of the auditory porus and CPA [41,42]. The most recent reports on this approach have been published in the last two years, confirming that ExpTTA is a safe and effective technique for the treatment of small vestibular schwannomas (Koos I, II and selected cases of Koos III) with low postoperative morbidity [43,44,45].

In 2018, the first case series of an exclusively transcanal endoscopic approach to treat lesions with limited extension at the suprageniculate fossa was reported. This suprageniculate endoscopic approach has been used since then for the complete removal of suprageniculate diseases (such as cholesteatoma, facial nerve schwannomas and haemangiomas) confined to an anatomical triangle formed by the middle cranial fossa (MCF), facial nerve and labyrinthine block, with low complication rates and in a minimally invasive fashion, although an ossiculoplasty may be necessary following disease removal for hearing rehabilitation [46]. In 2020, a large series on endoscopic and endoscopic-assisted approaches to the petrous apex was published, highlighting the increasing role of the endoscope in this kind of surgery [47]^.^

In the same years, the infracochlear approach was used for lesions located beneath the internal auditory canal, between the jugular bulb, the internal carotid artery (ICA) and the cochlea. This approach facilitates the removal of disease in the petrous apex below the IAC or vestibular neurectomy, while preserving hearing function [48,49].

Future advancements in endoscopic LSB surgery will likely depend on the spread of the endoscopic technique across otologic centres and its implementation in the training programmes of otology fellowships. Refinements in endoscopic surgical techniques and enhanced patient selection will influence the clinical applications and reports on these approaches. Technological advancements and the spread of alternative tools, such as the exoscope, will further define the future role of the endoscope in LSB surgery.

### 4.6. The Last Ten Years

**Figure 5:**
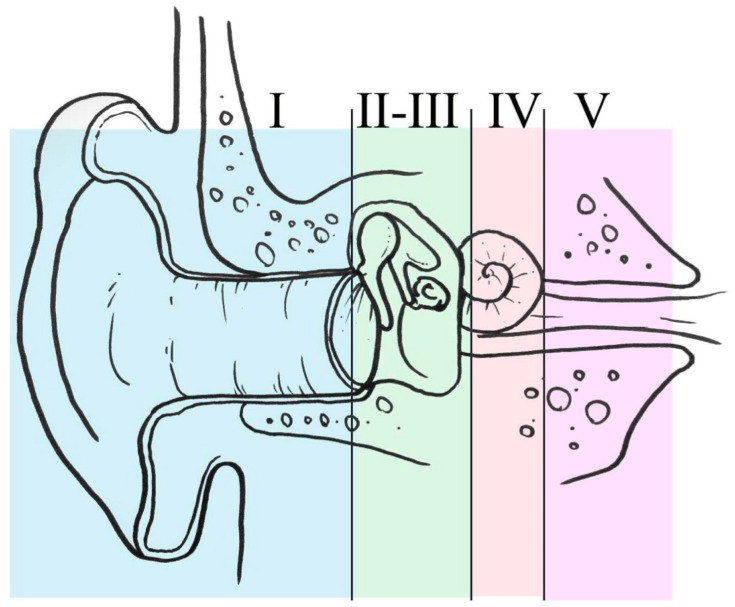
Five levels of training during EES. Level I: office-based procedures; level II: myringoplasties; level III: middle ear surgery; level IV: inner ear and geniculate area; level V: intradural pathology.

The last ten years have shown the most important popularization and spreading of endoscopic ear surgery all over the world. Several scientific events were focused on the subject, and also the issue of the training became a hot topic. A comprehensive training programme for middle ear and lateral skull base surgery, based on a progressive increase in complexity, was proposed [50]. Five stages of training were deemed appropriate for learning about endoscopic ear and lateral skull base surgery, ranging from simple middle and external ear procedures to surgery of the inner ear and internal auditory canal (Figure 5).

Mastering each level was suggested before attempting procedures at a higher level, in particular for procedures involving the lateral skull base. It was eventually postulated that adherence to such a programme could possibly decrease the rate of complications, making the training programme safer. Dissection courses started to introduce endoscopic techniques to their programmes, testifying that the modern ear and lateral skull base surgeon should master the entire spectrum of endoscopic and microscopic approaches, with the aim of tailoring the procedure and guaranteeing the best possible functional outcome. Step-by-step dissection courses were codified, including indications for the setup of the cadaver lab and the integration of the microscope and endoscope to enhance the use of both instruments [51].

Training on animal models became popularized, too, in particular on the ovine model [52]. The ovine model proved to be very useful, due to the anatomical similarities to the human middle ear. The main limitations described were the very long, bony external ear canal that could limit the manoeuvrability of the instruments, and also the absence of the mastoid. Nevertheless, most of the middle ear procedures could be performed on the ovine model, such as myringoplasties, stapes surgeries, t-tube placements and ossiculoplasties [53].

Some further expansions of the indications have also been verified in recent years, e.g., endoscopic facial nerve decompression [54]. The first pilot study reported a very high effectiveness in the treatment of post-traumatic facial palsy in patients with radiologic evidence of tympanic segment and/or perigeniculate region involvement. The approach introduced did not include any mastoidectomy or external incisions and proved to be very fast, visualizing in detail all the structures from the geniculate ganglion to the second genu of the facial nerve. In a comparative study, endoscopic facial nerve decompression resulted in early recovery, less postoperative pain and better postoperative air–bone gap closure when compared to conventional microscopic techniques [55]. The approach was further expanded to also decompress transcanally the labyrinthine portion of the facial nerve [56].

One of the crucial issues addressed in the last 10 years was bleeding control. Bleeding control in endoscopic ear surgery is particularly critical, due to the fact that the visualization tool (endoscope) has its tip in close contact with the surgical field, so it can often be dirtied by direct contact with blood, in particular when the bleeding is severe. For that reason, several techniques were codified for bleeding control, and the necessity for bleeding assessment during surgery arose. In 2017, a paper was published showing that the injection of diluted epinephrine (1:200,000, 2% mepivacaine), cottonoids soaked with epinephrine (1:1000), mono- or bipolar cautery, washing with hydrogen peroxide, and self-suctioning instruments could play an important role in bleeding control. The location of bleeding in the external auditory canal was most frequently in the posterior superior part, and inside of the middle ear, it was the pathology itself. This study confirmed that even the highest bleeding cases could be managed in an exclusively endoscopic technique [57]. In 2019, a dedicated scoring system named the Modena Bleeding Score (MBS) was introduced [58]. It provided five grades for rating the surgical field during endoscopic ear surgery procedures (from grade 1—no bleeding—to grade 5—bleeding that prevents every surgical procedure except those dedicated to bleeding control), proved to have high inter-rater and intra-rater reliability and was also used in further later studies to assess the results of interventional applications, even in endoscopic sinus surgeries [59]. For example, by the use of MBS, it was found that the data do not support a significant difference in bleeding conditions and hemodynamic parameters between EES patients receiving TIVA (total intravenous anaesthesia) and those receiving IA (inhalation anaesthesia) [60].

During the last ten years, consolidation of the results was testified to in the literature by the publication of comparative case series. In several instances, endoscopic cholesteatoma surgery has been shown to give similar or even better results in terms of recurrence or residuals [61,62], and less postoperative pain and in general a better quality of life [63], even in vestibular schwannoma surgery [64], if compared to the microscopic technique. The stability of the results in long-term follow-ups seemed confirmed for cholesteatoma surgery, too [65]. For stapes surgery [66,67,68], similar hearing improvements with both endoscopic and microscopic stapedotomy were shown, while the endoscopic approach showed advantages in reducing postoperative pain, dysgeusia and operative time. A non-inferiority performance was also confirmed in the paediatric population [69].

### 4.7. The Next Ten Years and Present Limitations

Although it is a relatively new technique, endoscopic ear surgery has had stunning improvements from previous years to the present day, also thanks to technological improvements [70]—but nonetheless, some limitations still persist. First of all, the use of a single hand makes the learning curve more difficult compared to other techniques. This is an issue that could be overcome in several ways, starting from the development and improvement of training. The popularization of 3D-printed temporal bone models suitable for endoscopic ear surgery [71] could make training more affordable, without losing the realistic features of surgical manoeuvring and anatomy [72]. Also, by the development of a dedicated virtual reality system, a safe and reliable way of training could be effectively improved. The use of a single hand could in future be overcome by the miniaturization of camera chips, which could reduce the dimensions and calibres of the endoscopes, thus increasing space to allow for three- or four-hand manoeuvres. Also, the improvement in knowledge of the regional vascular system, as well as the optimization of anaesthetic drugs, could be potentially helpful in bleeding control and better visualization, thus making the surgery and its learning easier. The introduction of a dedicated spreader of the external auditory canal, at present not available yet, could promote an increase in the room in the surgical field, and could potentially prevent dirtying of the tip of the endoscope. Other instruments dedicated to the intraoperative cleaning of the tip could help, in a similar way to what is already present in sinus surgery.

The introduction of different types of visualization devices, like steerable-tip endoscopes [73], could enable the enhanced accessibility of anatomical structures during TEES, which may lead to less extensive bone removal to facilitate minimally invasive TEES. Another limitation of endoscopic ear surgery at present is the two-dimensional visualization—the opposite of the three-dimensional visualization of the microscope. So, the introduction of 3D endoscopic systems would potentially allow for an even more realistic view of the surgical field compared to what is available nowadays.

Intraoperative systems that could help with a good degree of reliability to differentiate mucosal inflammation from cholesteatoma could also improve the residual disease rate in that pathology [74].

Not only technological improvements will represent the future of the endoscopic ear surgery. For instance, more clinical data about very long-term results (>10 years) are not available yet in the literature, despite being very important, in particular in cholesteatoma surgery. More studies focused on QoL are also needed on a big case series basis to definitely testify to the advantages of this minimally invasive technique compared to classic microscopic surgery. Also, multicentric studies are at present lacking: data from international cooperations of important otologic centres all over the world would be of paramount importance to reduce the biases related to single centres or national-based experiences.

## 5. Conclusions

Endoscopic ear surgery represented a true breakthrough in otology, having revolutionised concepts that were unchanged for decades. More improvements will follow in the coming years, and from being just an “adjunct” to traditional techniques, the endoscope has become and will become an even more fundamental tool in almost every otology procedure.
